# Determinants of root system architecture for future‐ready, stress‐resilient crops

**DOI:** 10.1111/ppl.13439

**Published:** 2021-05-07

**Authors:** Marco Lombardi, Laura De Gara, Francesco Loreto

**Affiliations:** ^1^ Department of Science and Technology for Humans and the Environment Campus Bio‐Medico University of Rome Via Alvaro del Portillo 21 Rome 00128 Italy; ^2^ Department of Biology, Agriculture, and Food Sciences National Research Council of Italy (CNR‐DISBA) Piazzale Aldo Moro 7 Rome 00185 Italy; ^3^ Department of Biology University Federico II via Cinthia Naples 80126 Italy

## Abstract

Climate change hampers food safety and food security. Crop breeding has been boosting superior quantity traits such as yield, but roots have often been overlooked in spite of their role in the whole plant physiology. New evidence is emerging on the relevance of root system architecture in coping with the environment. Here, we review determinants of root system architecture, mainly based on studies on *Arabidopsis*, and we discuss how breeding for appropriate root architecture may help obtain plants that are better adapted or resilient to abiotic and biotic stresses, more productive, and more efficient for soil and water use. We also highlight recent advances in phenotyping high‐tech platforms and genotyping techniques that may further help to understand the mechanisms of root development and how roots control relationships between plants and soil. An integrated approach is proposed that combines phenotyping and genotyping information via bioinformatic analyses and reveals genetic control of root system architecture, paving the way for future research on plant breeding.

## 1 INTRODUCTION—WHY BOTHER WITH ROOTS?

The growing population is in demand of crops able to meet the needs for food safety and food security, as well as crops with a reduced requirement of resources (high yield with limited availability of water, nitrogen, phosphate, and other mineral nutrients) (Lynch and Wojciechowski, 2015). The projected climate change scenarios show that the combination of increasing temperatures and reduction in precipitation distribution in time and space will lead to water stress by increasing evaporative demand in crops and will speed up soil degradation (Wheeler and von Braun, 2013). Plant breeding for desirable traits started thousands of years ago (Zeder, 2015), first by domesticating wild plants, and then by selecting plants with superior traits. Biotechnological knowledge led to a green revolution in the 1950s when Norman Borlaug and colleagues boosted quantitative traits such as yield (Swaminathan, 2009). However, breeding for yield only may have some negative consequences as well. Crops that grow fast and produce copiously are only sustainable in environments where natural resources are unlimited or supplemented by farming practices, with large impacts on soil fertility and biodiversity (Ribaudo *et al*., 2011). Under the current climate change scenario, however, breeding should reverse the old paradigm, selecting plants based on stress tolerance and resilience, and on traits that can be profitable both for mankind and the environment. Wild crop relatives that have survived changing environments and share genomes with domesticated plants may be the best genetic reservoir to look for genes conferring better adaptation to the environment, sustainable growth and production in harsh conditions, or improved constitutive or induced resistance to biotic and abiotic stresses (Preece and Peñuelas, 2020).

Not only have roots been rarely included in breeding programs, but breeding against a well‐developed root system has often been carried out, when selecting for high aboveground/belowground ratio biomass (Paez‐Garcia *et al*., 2015). Plasticity in root development and its role for stress tolerance is understudied, but the Food and Agriculture Organization of the United Nations encourages the development of breeding programs based on root system architecture (RSA) for more productive, sustainable, benign and resilient crops (Dubois, 2011). RSA accounts for the temporal and spatial distribution of roots in the soil, and thus determines the roots capacity to capture mobile and immobile resources (Lynch, 1995), primarily water and nutrients. Examining the published data, mainly collected in model plants such as *Arabidopsis*, we review how RSA can affect the plant's relationship with the soil, and how breeding for appropriate root architecture may help find plant phenotypes adapted to water scarcity and that are more resilient to the challenges of climate change.

### 1.1 RSA determinants

The main functions of the roots are: (1) to anchor plants; (2) to provide water and nutrients for plant growth; (3) to establish relationships with the flora and fauna of the soil, exploiting symbiotic ties; and (4) to store organic reserves. Supplying the plant with water and nutrients is by far the most essential function of the root system, a function that has been evolutionarily adapted to the water content available in the soil. If the roots do not provide enough water, the plant makes a series of physiological adjustments to limit the water loss through the leaves, by both dynamic (stomatal closure) and permanent (leaf shedding) behaviour. These adjustments have serious negative feedbacks for plant productivity, as CO_2_ acquisition by photosynthesis is also restrained, and part of the metabolic energy is devoted to synthesis of antioxidants counteracting the oxidative stress induced within cells or osmolites, thus increasing the capability to hold water (Denaxa *et al*., 2020; Per *et al*., 2017).

Here, we review different patterns of root trait development in plants, and will later focus on molecular rearrangements at the transcriptional, post‐transcriptional and translational level (Larkindale and Vierling, 2008) that might be responsible for phenotypic changes involving RSA, and might play significant roles for plant adaptation to water scarcity.

Different root systems characterise dicots and monocots. In dicots, primary roots (PRs), lateral roots (LRs) sometimes supported by adventitious roots (ARs), are the permanent root structure (Smith and de Smet, 2012). In monocots, shoot born roots overgrow the embryonic roots (PRs and LRs) and are responsible for most of the water and nutrient uptake (Koevoets *et al*., 2016).

**FIGURE 1:**
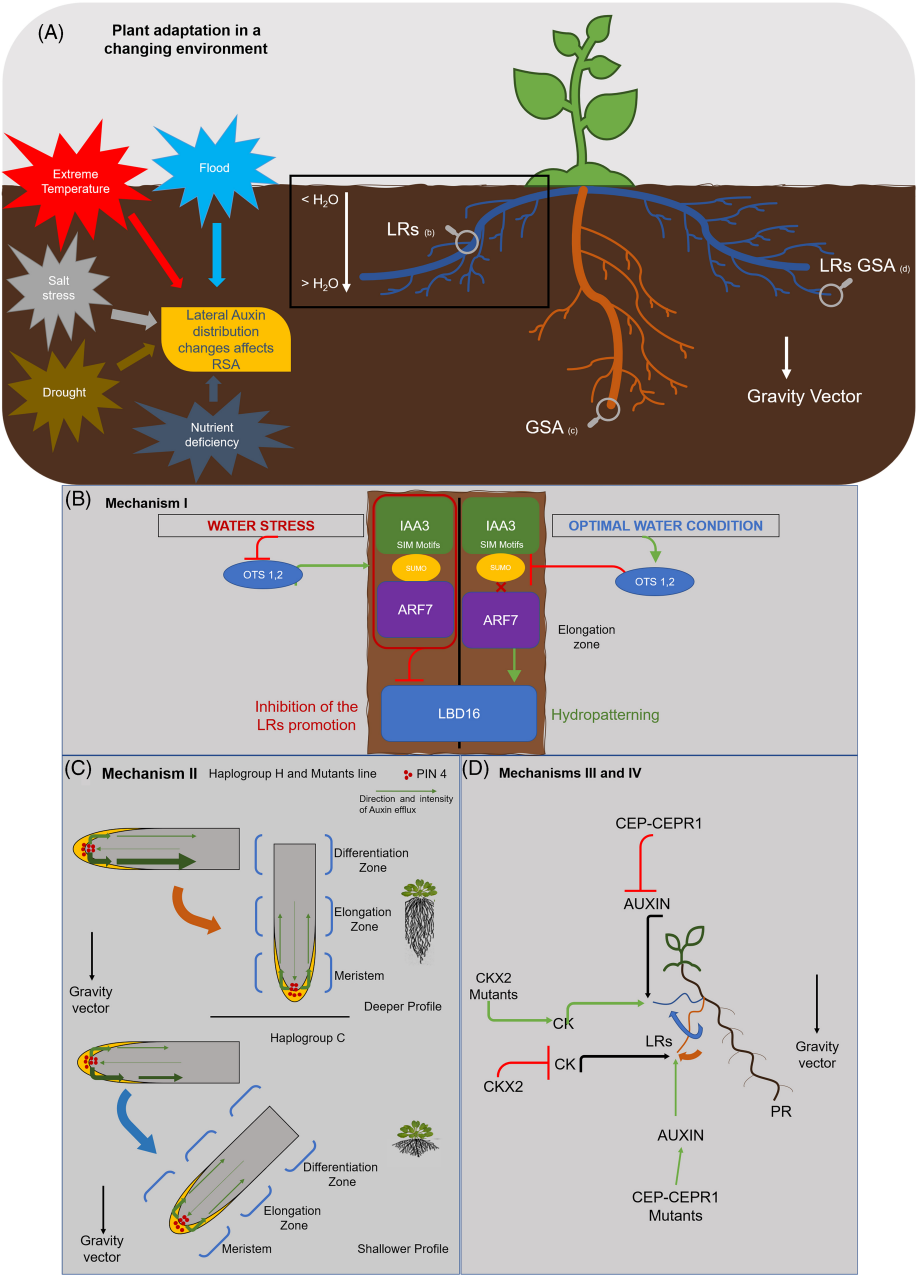
Molecular mechanisms driving root system architecture (RSA). (A) RSA is the phenotypic result of the interaction of genetic and environment controls (G × E)—Abiotic stresses (e.g. flooding; extreme temperatures; salt stress, etc.), often exacerbated by climate change, affect lateral auxin distribution via multiple molecular mechanisms, highlighted by the magnifier glasses, and elucidated in the following panels. (B) Root branching (lateral root [LRs] promotion) is shaped by mechanism I—Hydropatterning affects root branching in the elongation zone of the primary root (PR). Environmental condition, i.e. water stress and optimal water conditions affect SUMO proteases (OTS1,2). On the dry side of the root (on the left of Panel B), water stress inhibits SUMO proteases, in turn allow the formation of the repressor complex (red circle) (IAA3‐SUMO‐ARF7), thereby blocking (red arrow) auxin‐responsive gene expression associated with LR initiation. On the right of Panel B (optimal water conditions), moisture inhibits (red arrow) the formation of the repressor complex (IAA3‐SUMO‐ARF7) affecting SUMO. Thus, ARF7 induced asymmetric expression of *LBD16* in LRs. Light green arrow: activation; red arrow: inhibition; red cross: missing contact between ARF7 and SUMO. (C) Gravitropic set‐point angle (GSA) is shaped by mechanism II—Green arrows show the direction and intensity of auxin efflux via PIN4 (red points). The exocyst factor, *EXO70A3*, modulate the asymmetric auxin gradient between the lower and upper side of the root tip via PIN4 proteins. Gravity stimulus in columella cells sets the direction of the root bending according to the allelic variation of *EXO70A3* determining the threshold of auxin flux. Thus, two models of extreme wild‐type phenotypes resulting in a deeper RSA profile (upper side) and in a shallower RSA profile (lower side) are proposed. Shallower RSA (in blue) is shared by haplogroup C and guarantees exploration of the soil more shallower layers and access to superficial water by herbaceous crops. However, some selected varieties, like haplogroup H, root deeply (in orange). (D) GSA of LRs is shaped by mechanisms III and IV—CEP‐CEPR1 and cytokinin (CK) signalling are proposed as molecular mechanism shaping LRs GSA during the III stage. CEP‐CEPR1 signalling inhibits both rootward auxin transport and shoot auxin levels, in turn affecting bending of GSA of the LRs and inducing a shallower root profile (blue arrow). Auxin flux that affects GSA of the LRs is balanced by CK that act as anti‐gravitropic signal at opposing organ flanks. CK oxidase 2 (*CKX2*) affects CK pathway, its natural allelic variation shapes different degree of LRs bending. *CKX2* mutants and wild type reflect two opposite phenotypes respectively showing a shallower and deeper LRs profile. Light green arrow: activation; red arrow: inhibition; black arrow: a consequence of a previous activation or inhibition

Both in dicots and monocots, auxins play a key role in determining root architecture. Auxins are important hormones and ubiquitous regulators of almost every component of plant development (Chapman *et al*., 2020). Auxins affect the development of PRs, ARs, LRs, and also control root angle relative to the gravity vector, termed the gravitropic set‐point angle (GSA) (Wang *et al*., 2015). GSA is a key regulator of RSA exploration of the soil, characterised by deeper or shallower root profiles (Figure 1). GSA of LRs plays a role in the adaptation aimed to face environments which are scarce of resources. Deeper RSA allows access to water and nutrients stored deeper in the soil (Wasson *et al*., 2012). Deep rooting and LRs have been proposed as a key trait of agronomical interest (Ferguson, 2019).

Gravitropic response to vector gravity is perceived by the columella cells in the root tip, but the response occurs in the elongation zone. LRs emergency and growth is shaped through four stages. At Stage I, LRs emerge in the main root at a 90° angle from the vector of gravity, and is followed (Stage II) by root bending with a defined and highly controlled GSA (Rosquete *et al*., 2013). In Stage III, LRs grow away from the PR thus defining the initial GSA, until a new gravitropic response is expressed and a new bending takes place (Stage IV) (Rosquete *et al*., 2018).

### 1.2 RSA molecular mechanisms

RSA phenotypes have recently been associated with the molecular mechanisms controlling root growth, GSA, and in particular GSA of LRs (Figure 1A). The following mechanisms, at least in *Arabidopsis*, have received compelling experimental support so far and are correlated to main root traits: (1) hydropatterning response (Orosa‐Puente *et al*., 2018) (Figure 1B); (II) PIN 4 expression (Figure 1C) (Ogura *et al*., 2019); (III) cytokinin (CK) signalling (Waidmann *et al*., 2019); and (IV) C‐terminally encoded peptides (CEP)–CEP receptors 1 signalling (Chapman *et al*., 2020) (Figure 1D). Interestingly, all the above‐mentioned mechanisms are based on modifications of the auxin‐dependent patterns of root responses to gravity.

Hydropatterning, was postulated by Orosa‐Puente *et al*., (2018) when observing that root branching occurs when roots get in contact with moisture in the elongation zone (Figure 1B). A family of transcription factors termed auxin response factors 7 (ARF 7) induce asymmetric expression of its target gene *LBD16* in LRs founder cells. The experiment was conducted in Petri plates mimicking in vitro vertical growth, i.e. with roots expanding through a medium and exposed at opposing sides to either air or moisture. In the air side of the root, small ubiquitin‐like modifier (SUMO) proteins recruit the Aux/IAA protein (IAA3) and create a transcriptional repressor complex that negatively regulates ARF7 DNA binding activity, thereby blocking auxin‐responsive gene expression associated with LR initiation. On the wet side of the root, SUMO protease inhibits the formation of the repressor complex activating *LBD16* genes.

Mechanism II was revealed recently by Ogura on *Arabidopsis* growing in dry environments (Ogura *et al*., 2019). *Arabidopsis thaliana* is the main dicot model organism due to its fast growth that allows rapid phenotypic characterisation, and the complete knowledge of its genetic background (Zhu *et al*., 2016). *Arabidopsis*' natural accessions were classified by Ogura *et al*., (2019) into two haplogroups characterised by common (C) and high (H) response of root growth direction (horizontality) to the auxin transport inhibitor *N*‐1‐naphtylphthalamic acid. The mechanism is based on the expression of PIN4 proteins in columella cells controlled by the *EXOCYST70A3* gene, which modulates auxin transport, in turn shaping the root GSA in the soil (Figure 1C). As a consequence of allelic variations of *EXOCYST70A3*, haplogroup H has deep roots, while haplogroup C has shallow roots. Haplogroup C was proposed to confer a mechanism of adaptation to environments with temporally limited water availability. Indeed, shallow root architecture shows better drought tolerance under variable rainfall patterns in these *Arabidopsis* plants (Ogura *et al*., 2019).

Mechanism III suggests that root exploration of the soil by LRs depends on CK concentration (Figure 1D). Waidmann *et al*., (2019) showed that CK signalling affects root gravitropic growth. In particular, asymmetric CK signalling reduces growth at the upper side of recently emerged LRs, but not in PRs. Thus, CK signalling plays a developmental role in establishing the primary GSA of LRs. Natural variations of the *cytokinin oxidase 2 (CKX2)* gene in *A*. *thaliana* accessions depends on a single nucleotide polymorphisms (SNPs) causing a single amino acid alteration where isoleucine is replaced by methionine. CKX enzymes are responsible for the irreversible degradation of CK via the oxidative cleavage of their side chain, thus blocking CK accumulation. The inactive *CKX2* variant shared by specific *Arabidopsis* accessions, besides allowing better acclimation to extreme winters (Xiao and Zhang, 2020), promotes a shallower RSA and seems to be a promising trait to cope with hypoxic stress (Waidmann *et al*., 2019). Indeed, CK signalling plays an important role in adaptation to hypoxia. The inactive *CKX2* variant experienced in natural accessions adapted to extreme environments also provides a significant chance to survive under oxidative stress (Xiao and Zhang, 2020).

In Mechanism IV, Chapman *et al*. (2020) suggested that shoot‐located CEP–CEP receptors 1 signalling may control the GSA of LRs in Stage III. The CEP‐hormone interaction inhibits the auxin pool size and rootward auxin transport, increasing the GSA of LRs (making LRs shallower). Disruption of CEP‐CEPR1 signalling results in a deeper RSA (Figure 1D—orange arrow).

It is still unclear whether all four mechanisms contribute to shaping RSA and setting GSA in plants. *Arabidopsis* is surely a gold standard model, and the availability of natural ecotypes will provide further targets for molecular breeders aiming to manipulate the crop's RSA. However, whether the findings concerning RSA obtained with simplified laboratory conditions can be confirmed when plants grow in real soil and in the field is still widely debated (Rich and Watt, 2013). RSA is influenced by many soil constraints, such as mechanical strength, the density of the soil air pockets, soil pH, temperature and heterogeneity, as well as by biological interactions with soil flora and fauna. All these conditions may further impact root phenotyping, perhaps beyond modulating the mechanisms already elucidated (Smith and de Smet, 2012). Moreover, root phenotyping in field conditions has been difficult because high‐throughput and non‐destructive systems able to visualise RSA have not been available until recently (Fiorani and Schurr, 2013).

### 1.3 RSA: Further insights by combining genotyping and phenotyping information, bioinformatic analyses, and new biotechnological tools

Nowadays, phenotypic consequences of genetic manipulation can be observed working in a pipeline that integrates new genotyping and phenotyping technologies, thus contributing to establish which mechanisms shape RSA under the different environmental conditions. While predicting how traits affect RSA in time and space under varying conditions is now possible, this requires: (1) high‐throughput and non‐destructive measurements of as many root traits as possible; (2) mathematical modelling approaches to clearly identify and separate suitable phenotypes among available natural biodiversity; and (3) further novel plant breeding technologies for precise introduction of the suitable genotypic traits in target plants with an impact on economics. We now review the latest tools that may be suitable to detect/confirm genetic control of RSA and to assess RSA impact on plant growth and production.

#### 1.3.1 Phenotyping

**FIGURE 2:**
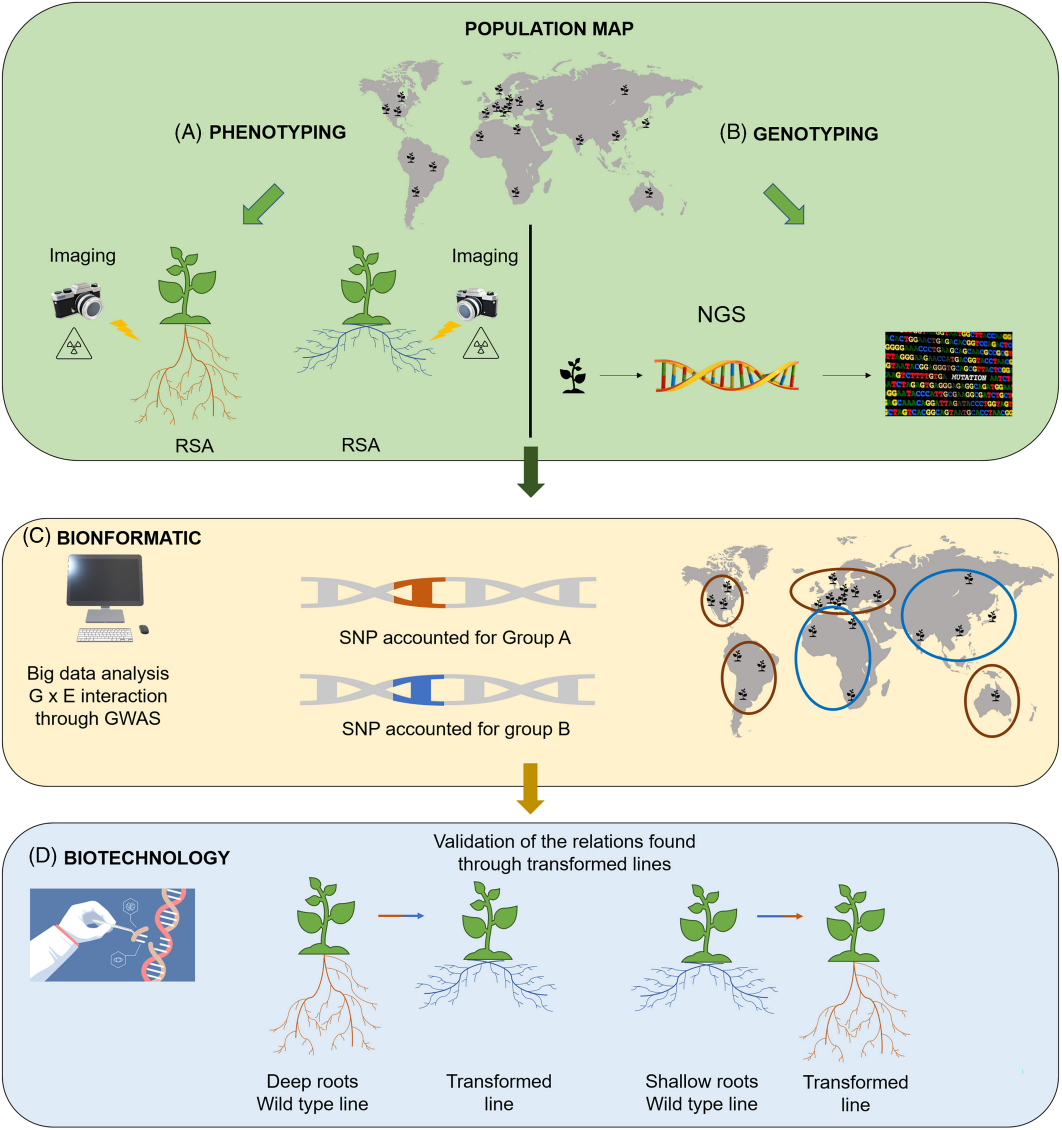
Integrated approach for a population mapping study. To extract data about root system architecture (RSA) within a population requires high throughput phenotyping and genotyping analysis. The main target of new research facilities is to follow root growth in natural condition (in soils) to obtain a realistic RSA profile with no destructive approaches and no invasive technologies. Hi‐tech core facilities and advanced knowledge, i.e. X‐ray micro‐computed tomography (μCT) for phenotyping and next‐generation sequencing (NGS) for genotyping, are promising skills for future scientific research. The grey map is just an example aiming to show the casual distribution of a plant species (e.g. *Arabidopsis*), with natural accessions adapted to different climate and soil conditions worldwide. Indeed, natural accessions among a population map provide both phenotypic (A) and genotypic (B) variation of RSA traits. (C) Merging phenotypic and genotypic data with the help of bioinformatic techniques, as Genome Wide Association Studies (GWAS), may lead to new correlation, i.e. SNP accounting for similar RSA trait phenotypes across the genetic diversity sampled. In the example of Figure 2, two SNPs accounted for one Group A phenotype (deep rooting) and one Group B phenotype (shallow rooting), respectively, represented with brown and blue circles over the planet. Thus, the map is an example that would represent the distribution of a phenotype of a plant species. (D) The use of transgenic lines with biotechnology approaches, i.e. CRISPR Cas‐9, are pivotal for the validation of relation found with GWAS. To characterise the genes involved in the shaping of the phenotype, transformed lines are used where the identified genes are knocked down or overexpressed to revert the root phenotype observed in the wild‐type lines

Recent advances in phenotyping finally made it possible to display root structure and enable imaging of PR length and other more complex root traits such as GSA, bushiness, root distribution in different zones, diameters of different root parts, convex hull, or root volume (Deja‐Muylle *et al*., 2020) (Figure 2A). The cluster of these non‐destructive analyses is composed of 2D techniques that range from seedlings maintained in agar plates up to whole plants in specialised rhizotrons. More recently, sophisticated 3D techniques as X‐ray micro‐computed tomography (X‐ray μCT), based on multiple‐viewpoint imaging of plants grown in optical media have also been used (Clark *et al*., 2011).

X‐ray μCT scanning system is a versatile non‐destructive 3D approach for observations of plant root architecture and soil structural properties with the support of advanced imaging techniques. Attenuation of interaction of X‐ray with the sample is analysed, allowing the acquisition of high quality and contrast low noise radiograph images, which also allows fast scan times (Mairhofer *et al*., 2015). For X‐ray μCT measurements no special sample preparation is necessary, and the environmental conditions within the chamber are easily kept stable during the scanning process. The scanning facility permits examination and quantification of emerging root architecture in soil systems over time. Following scanning, roots are imaged using Volume Graphics software and RSA quantification tools such as RooTrak (Mairhofer *et al*., 2017). RooTrak is a software that views μCT data as a sequence of images through which root objects appear to move as the x‐y cross sections are traversed along the z‐axis of the image stack (Mairhofer *et al*., 2012). RooTrak can successfully extract a range of root architectures from the surrounding soil and promises to facilitate future root phenotyping efforts. The main shortcomings of the μCT are the functional complexity of the system, the high cost and the need for highly specialised laboratory managers and operators. Progress in technologies and materials should rapidly lessen such constraints in the future. Other 3D imaging techniques are also available to analyse in situ root development such as magnetic resonance imaging and nuclear magnetic resonance (Mooney *et al*., 2012). Merits and limitations of such techniques (often very similar to those highlighted for μCT) have recently been reviewed (Atkinson *et al*., 2019).

#### 1.3.2 Integrated approach: Genome wide association studies of large populations with different RSA phenotypes

Next‐generation sequencing, also known as high‐throughput sequencing (Figure 2B), describes DNA sequencing technologies that enable the collection of big data about genetic information (Behjati and Tarpey, 2013). By comparatively analysing the genome and transcriptome of the phenotypes, differences can be detected that may reveal the involvement of one or more of the known mechanisms, their complementary effects, or additional mechanistic explanation for the observed traits and whole RSA. Access to a bioinformatics platform (Figure 2C) as Genome Wide Association Studies (GWAS), allowing handling and processing of big data, will provide invaluable insight into the putatively highly non‐linear interactions between genomic information and root phenotypes (Atkinson *et al*., 2019). GWAS, in particular, is the latest and most powerful tool to identify genetic variations between populations of organisms, as is the case for the natural accessions of *A*. *thaliana* with a wide shape of RSA phenotypes (Menguer *et al*., 2017). The genetic variation of candidate traits is driven by a suite of quantitative trait loci or in some cases by SNPs. Essentially, GWAS studies use large populations to quantitatively evaluate the likelihood of an association between variants and phenotypic outcomes (Balding, 2006). The characterisation of the screened gene and validation of the relations found between genes and functions, is confirmed by the generation of transgenics lines overexpressed and/or knocked‐out by genome editing, i.e. CRISPR‐Cas (Figure 2D). Genes underpinning crops traits can reveal a resilient phenotype and therefore contrast the overall impact of abiotic stresses. This new information can be the starting point for the creation of new resilient crops. In order to preserve soils for the next generations, future studies should take into account New Breeding Techniques (NBTs) methods to further integrate results obtained with model plants and on crops, focusing on those traits that confer yield stability and resilience to stresses. Indeed, NBTs could increase and accelerate the development of new traits in plant breeding by several techniques, including, e.g. site‐directed nucleases (i.e. CRISPR‐Cas9 system), oligonucleotide directed mutagenesis, cisgenesis end intragenesis, RNA‐dependent DNA methylation, grafting (on GM rootstock), reverse breeding, agro‐infiltration (Lusser *et al*., 2011), or directed evolution (Gionfriddo *et al*., 2019).

## 2 CONCLUSIONS AND FUTURE PERSPECTIVES

Better exploitation of soil resources including water and mineral acquisition by plant RSA may improve sustainable use of land by agriculture (Kell, 2012), and this is finally directing scientific attention to the often disregarded role of roots and in general to belowground biology (Preece and Peñuelas, 2020). The search for the molecular mechanism(s) shaping RSA traits is benefiting from new tools that may soon provide new answers to our questions about root physiology. Evidence collected so far points out that auxin concentration and movement may play a major role in setting the RSA, but other mechanisms may be equally important or also contribute to root phenotypes in nature and in farming soils.

For example, the interaction with soil‐borne organisms, can affect root growth, via signalling of selected volatile organic compounds (VOCs). The biochemical and molecular mechanisms underlying these very interesting interactions at the plant phenome level are still to be unravelled. However, Moisan *et al*. (2021) showed that the timing and duration of exposure to VOCs produced by the soil‐borne fungus *Rhizoctonia solani* affect the RSA phenotype of *Brassica rapa*.

An additional level of complexity can also involve reactive oxygen species (ROS), known for being involved in controlling both developing and stress responsive pathways (Locato *et al*., 2018). Root meristem growth factor 1 (RGF1) is a peptide hormone that controls root meristem size through ROS signalling (Yamada *et al*., 2020). Although the downstream molecular pathway remains unknown, the work of Yamada et al. demonstrated that RGF1 controls the distribution of ROS along with the roots of *A*. *thaliana*. This evidence further underlines how several developmental and environmental players crosstalk in defining root growth, in addition to the genetic programs characterizing each species or ecotypes.

RSA might also be controlled by different mechanisms reflecting plant evolution. As seen above, monocots and dicots do not share a similar root system. Consequently, monocots with fibrous roots might not be controlled by the same mechanisms that set RSA in dicots with long and deep taproots. As also mentioned, a superficial root system would allow *Arabidopsis* to fully capture water made available by brief seasonal rains in dry environments (Pandey and Bennett, 2019). This model supports previous observations on grasses (Hartnett *et al*., 2013). However, it is an established and old notion that deeper roots allow crops to exploit fresher and more profound water‐rich soil volumes under drought condition. As an example, *DEEPER ROOTING 1* gene expression enhances rice yield under drought stress by setting steeper and deeper GSA (Uga *et al*., 2013). *Arabidopsis* and annual, short‐lived herbs may have a different strategy of foraging in the soil that might depend on the plant acclimation to the environment.

There is a need to move from model plant species like *A*. *thaliana* to traded crops that may actually benefit from phenotypes with root systems that fit the environment, therefore providing plants which are climate‐ready, productive, and efficient in using soil and water resources.

However, how transferable the results obtained in model plants are to crops is still unclear. More information on root phenotypes of crop varieties adapted to different environments, or on the RSA of the wild types from which modern crops derive, could improve our capability to identify genetic patterns useful for breeding and representing a bottleneck for plant productivity and resilience.

Both in *Arabidopsis* and crops, different GSAs are associated with specific abiotic stress avoidance and to an allelic variation of natural accessions. These models are the result of adaptation over the years. Further understanding could enable the breeding of crop cultivars that are suitable for the different stress conditions (Rogers and Benfey, 2015). Some crops may be particularly suitable for this “tailored” approach. Major monocot cereals that are grown worldwide and provide 60% of the human calories are now studied extensively for their RSA at major phenotyping facilities worldwide (Atkinson *et al*., 2019; Smith and de Smet, 2012), and the genome of these species is also largely known and available, but environmental impacts on the expression of genes controlling root development are still understudied (Ferrero‐Serrano and Assmann, 2019). In other cases, farming has already naturally selected for phenotypes with favourable agronomic RSA traits, as exemplified by the case of wine grape rootstocks with different rooting angle and distribution, depending on climate and soil conditions of growth (Ollat *et al*., 2014). In this case, genotypic information is often still missing, but matching genome and phenome should not be overly complicated and should yield important scientific information for further plant breeding. Manipulation experiments reproducing future climatic scenarios will also be instrumental in tailoring root systems that allow better soil conservation and use, and contribute to achieving United Nation Sustainable Development Goals such as Zero Hunger and Life on Lands (https://sdgs.un.org/goals/goal2).

## Data Availability

Data sharing is not applicable to this article as no new data were created or analyzed in this study.
